# Impact of Adjuvant Chemoradiotherapy on Survival of Resected Pancreatic Adenocarcinoma Cancer: A Surveillance, Epidemiology and End Results (SEER) Analysis

**DOI:** 10.3389/fonc.2021.651671

**Published:** 2021-07-01

**Authors:** Xiaomao Shi, Jin Peng, Huangang Jiang, Yu Gao, Wenbo Wang, Fuxiang Zhou

**Affiliations:** ^1^ Department of Chemotherapy and Radiation Therapy, Zhongnan Hospital, Wuhan University, Wuhan, China; ^2^ Hubei Key Laboratory of Tumor Biological Behaviors, Zhongnan Hospital, Wuhan University, Wuhan, China; ^3^ Department of Chemotherapy and Radiation Therapy, Hubei Key Laboratory of Tumor Biological Behaviors, Zhongnan Hospital of Wuhan University, Wuhan, China

**Keywords:** adjuvant therapy, chemoradiotherapy, chemotherapy, pancreas, radiotherapy

## Abstract

**Background:**

The benefits of postoperative adjuvant chemoradiotherapy (CRT) for pancreatic cancer remain controversial. The purpose of this study is to determine if adjuvant CRT can improve the overall survival of postoperative pancreatic cancer patients compared to adjuvant chemotherapy (CT).

**Methods:**

Patients with resected pancreas adenocarcinoma were identified in the Surveillance, Epidemiology, and End Results (SEER) database (2004–2016). Multivariate Cox regression was used to determine the factors related to survival rate. Selection bias was reduced to a minimum through propensity matching analysis. Subgroup analyses by clinical characteristics were performed.

**Results:**

This study identified 10,097 patients who received adjuvant CT (n = 5,454) or adjuvant CRT (n = 4,643). On multivariate analysis, age, sex, tumor size, site, grade, stage, T stage, and lymph node metastasis were independent risk factors for OS. The basic clinical characteristics were well balanced after propensity matching. After propensity matching, CRT can improve the survival rate compared with CT [median OS: 22 months *vs* 23 months (HR, 0.928; 95% CI, 0.881–0.977; P = 0.004)]. Subgroup analysis indicated that the survival benefit of adjuvant chemoradiotherapy was more significant in patients with female (HR, 0.860; 95% CI, 0.798–0.926; P = 0.005 for interaction) or T3 (HR, 0.905; 95% CI, 0.855–0.957; P = 0.04 for interaction) or lymph nodes positive (HR, 0.883; 95% CI, 0.832–0.938; P = 0.005 for interaction).

**Conclusion:**

Adjuvant CRT was associated with improved survival compared with adjuvant CT in patients with resection of pancreatic ductal adenocarcinoma. The benefit was more significant in patients with female or T3 or lymph nodes positive.

## Background

Pancreatic cancer is one of the most aggressive tumors. It is the seventh leading cause of cancer deaths among men and women worldwide, with approximately about 459,000 new cases and 432,000 deaths, according to GLOBOCAN 2018 estimates (1). The 5-year overall survival rate (OS) is 5 to 20%. The vast majority of patients with pancreatic cancer present as locally advanced unresectable tumors at the first diagnosis, and their clinical treatment is complicated and the prognosis is poor. Only 10 to 20% of patients are resectable at the time of initial diagnosis (2–5). Although surgical resection is performed, most patients have local recurrence or distant metastasis within 2 years (6–8), so postoperative systemic and local adjuvant therapy after surgery is particularly important. Multiple studies have confirmed the survival benefits of adjuvant CT in pancreatic cancer (9–12). However, many studies on adjuvant CRT have not reached a consistent conclusion (11, 13–15). The reason for the contradiction may be that the lack of a standardized plan for adjuvant CRT leads to defects in the design and implementation of early clinical trials, as well as the use of antiquated techniques (16–19).

In this retrospective analysis based on the SEER database, we evaluated whether adjuvant CRT improved survival in patients undergoing resection of pancreatic cancer. Propensity matching analysis was used to minimize the confusion caused by non-random treatment allocation. At the same time, we conducted a subgroup analysis of patients to identify subgroups in which CRT has potential or significant benefits compared with adjuvant CT.

## Methods

### Patients

We retrospectively studied the survival outcomes of adjuvant therapy in patients with pancreatic cancer diagnosed in the SEER database. We identified patients with non-metastatic pancreatic adenocarcinoma who underwent surgical resection between 2004 and 2016. Patients who received adjuvant CT or adjuvant CRT after surgery were included. Neuroendocrine tumors and other histological types were excluded. Patients who received neoadjuvant therapy, unknown lymph node status, the survival time of less than 90 days, and less than 40 years old were excluded. We incorporate clinical features including age, sex, race, primary site, tumor size, tumor differentiation, tumor stage, T staging, lymph node status, and type of adjuvant treatment.

### Statistical Analysis

The Chi-square test or Fisher’s exact test were used for the analysis of categorical variables. Survival estimates were calculated using Kaplan–Meier survival curves and compared using the log-rank test. Univariable and multivariable regression analyses were performed in all patients using a Cox proportional hazard model. To manage the confounding factors of treatment selection bias, a propensity score-matched analysis was carried out. To perform 1:1 nearest neighbor propensity score matching (PSM) without substitution. The balanced distribution of two groups of matched patients was verified by the chi-square test for classified variables. All statistical tests were two-sided, and a P value <0.05 was considered statistically significant. All statistical analyses and graphics were performed using the software SPSS Statistics (IBM, Armonk, NY, USA) and the statistical software package R version 4.0.1.

## Results

We included 10,097 patients with pancreatic adenocarcinoma who met the criteria. A majority of patients aged 60 to 79 years (65.6%) and 51% were male. Most patients had T3 tumors (78.1%), were stage II (85.9%), and had nodal involvement (67.5%) and moderately differentiated tumor (48.3%). Most of the tumors occurred in the head of the pancreas (73.6%), and most of them were between 2 and 4 cm (58.3%) in size. Of these patients, 5,454 (54%) received adjuvant CT and 4,643 (46%) received adjuvant CRT. When the patients receiving adjuvant CT and adjuvant CRT were compared, there was a significant difference in age, gender, and tumor size, tumor differentiation, T stage, and lymph node status among the patients. **Table 1** displays the demographic and clinical characteristics.

**Table 1:** Demographic and clinicopathological features of patients receiving adjuvant chemotherapy and adjuvant chemoradiotherapy.

Variable	Adjuvant Chemotherapy (n = 5,454)	Adjuvant Chemoradiotherapy (n = 4,643)	*P* Value
Age			<0.001
40–59	1,378 (25.3%)	1,442 (31%)	
60–79	3,637 (66.7%)	2,989 (64.4%)	
>80	439 (8%)	212 (4.6%)	
Sex			0.007
Female	2,738 (50.2%)	2,206 (47.5%)	
Male	2,716 (49.8%)	2,437 (52.5%)	
Race			0.162
White	4,520 (82.9%)	3,817 (82.2%)	
Black	503 (9.2%)	478 (10.3%)	
Other	431 (7.9%)	348 (7.5%)	
Primary Site			0.015
Head	3,952 (72.5%)	3,476 (74.9%)	
Body/tail	1,009 (18.5%)	763 (16.4%)	
Other	493 (9%)	404 (8.7%)	
Grade			<0.001
1	541 (10%)	432 (9.3%)	
2	2,522 (46.2%)	2,350 (50.6%)	
3	1,910 (35%)	1,552 (33.4%)	
4	65 (1.2%)	42 (0.9%)	
Unknown	416 (7.6%)	267 (5.8%)	
Tumor Size			0.018
≤2 cm	929 (17%)	697 (15%)	
2–4 cm	3,156 (57.9%)	2,726 (58.7%)	
>4 cm	1,369 (25.1%)	1,220 (26.3%)	
Stage			<0.001
I	610 (11.2%)	364 (7.8%)	
II	4,612 (84.6%)	4,061 (87.5%)	
III	232 (4.2%)	218 (4.7%)	
T			<0.001
T1	344 (6.3%)	179 (3.9%)	
T2	670 (12.3%)	564 (12.1%)	
T3	4,208 (77.2%)	3,682 (79.3%)	
T4	232 (4.2%)	218 (4.7%)	
Nodal status			<0.001
N0	1,877 (34.4%)	1,402 (30.2%)	
N1	3,577 (65.6%)	3,241 (69.8%)	

CRT, chemoradiotherapy.

### Association of Adjuvant Chemoradiotherapy With Survival

**Figure 1:**
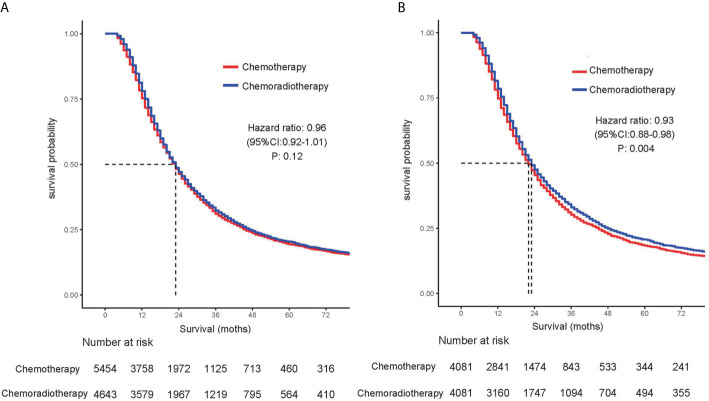
Comparison of overall survival for patients treated with chemotherapy alone and patients treated with chemoradiotherapy in **(A)** unmatched and **(B)** propensity score–matched analyses.

**Table 2** summarizes the univariate and multivariate analyses of factors associated with overall survival. Univariate Cox proportional hazards modeling analysis showed that age, gender, race, and tumor size, primary site, tumor differentiation, and stage, T stage, and lymph node status were correlated with survival. The significant correlation variables of univariate analysis were incorporated into multivariate Cox regression. In multivariate analysis, there was no statistical difference between race and OS, and other variables were still statistically significant. For the overall cohort, median survival was 23 months. In the cohort before propensity matching analysis, adjuvant CRT did not show survival benefits compared with adjuvant CT [median OS: 23 months *vs* 23 months (HR, 0.964; 95% CI, 0.920–1.010; P = 0.123)] (**Figure 1A**).

**Table 2:** Univariate and multivariate analysis of overall survival rate.

Variable	Univariate			Multivariate		
	HR	95% CI	*P* Value	HR	95% CI	*P* Value
Age			<0.001			<0.001
40–59	1			1		
60–79	1.094	1.037–1.153	0.001	1.101	1.044–1.161	<0.001
>80	1.261	1.140–1.394	<0.001	1.271	1.148–1.408	<0.001
Sex						0.002
Female	1			1		
Male	1.086	1.037–1.138	0.001	1.078	1.028–1.130	
Race			0.031			0.109
White	1			1		
Black	1.035	0.958–1.119	0.383	1.081	0.999–1.169	0.053
Other	0.894	0.815–0.980	0.016	0.970	0.884–1.064	0.516
Primary Site			<0.001			0.009
Head	1			1		
Body/tail	0.866	0.812–0.923	<0.001	0.905	0.847–0.967	0.003
Other	0.935	0.860–1.016	0.114	0.943	0.866–1.026	0.172
Grade			<0.001			<0.001
1	1			1		
2	1.375	1.259–1.502	<0.001	1.295	1.185–1.415	<0.001
3	1.833	1.675–2.006	<0.001	1.660	1.516–1.818	<0.001
4	1.498	1.172–1.914	0.001	1.505	1.177–1.923	0.001
Unknown	1.232	1.086–1.398	0.001	1.232	1.085–1.399	0.001
Tumor Size			<0.001			<0.001
≤2 cm	1			1		
2–4 cm	1.421	1.325–1.524	<0.001	1.246	1.150–1.350	<0.001
>4 cm	1.675	1.550–1.809	<0.001	1.470	1.347–1.606	<0.001
Stage			<0.001			<0.001
I	1			1		
II	2.062	1.882–2.258	<0.001	1.269	1.111–1.451	<0.001
III	3.204	2.796–3.671	<0.001	2.390	1.981–2.884	<0.001
T			<0.001			0.027
T1	1			1		
T2	1.540	1.340–1.769	<0.001	1.064	0.899–1.239	0.445
T3	2.091	1.847–2.368	<0.001	1.180	1.000–1.371	0.040
T4	3.224	2.478–3.784	<0.001	2.390	1.981–2.884	<0.001
Nodal status						<0.001
Negative	1			1		
Positive	1.661	1.576–1.750	<0.001	1.441	1.358–1.530	
Adjuvant therapy			0.124			0.003
Chemotherapy	1			1		
Chemoradiotherapy	0.964	0.920–1.010		0.931	0.888–0.976	

CI, confidence interval.

### The Influence of Propensity Matching on Survival

Before the entire queue is matched, there is an imbalance in most of the variables between the two groups. Therefore, to build a well-balanced queue, the confounding factors are controlled by propensity matching. The independent factors related to OS, such as age, sex, tumor size, location, grade, stage, T stage, and lymph node metastasis, were matched at 1:1. **Table 3** shows the baseline characteristics after matching, and the difference between the two groups has been balanced. The median survival of the tendentious matching queue is 23 months. Patients who received adjuvant CRT had an advantage in survival compared with patients receiving adjuvant CT [median OS: 22 months *vs* 23 months (HR, 0.928; 95% CI, 0.881–0.977; P = 0.004)] (**Figure 1B**).

**Table 3:** Demographic and clinicopathological features of patients receiving adjuvant chemotherapy and adjuvant chemoradiotherapy after propensity score matching.

Variable	Adjuvant Chemotherapy (n = 4,081)	Adjuvant CRT (n = 4,081)	*P* Value
Age			0.976
40–59	1,126 (27.6%)	1,125 (27.6%)	
60–79	2,771 (67.9%)	2,776 (68%)	
>80	184 (4.5%)	180 (4.4%)	
Sex			0.877
Female	1,978 (48.5%)	1,971 (48.3%)	
Male	2,103 (51.5%)	2,110 (51.7%)	
Primary Site			0.970
Head	3,131 (76.7%)	3,125 (76.6%)	
Body/tail	647 (15.9%)	655 (16%)	
Other	303 (7.4%)	301 (7.4%)	
Grade			0.963
1	346 (8.5%)	337 (8.3%)	
2	2,081 (51%)	2,086 (51.1%)	
3	1,421 (34.8%)	1,423 (34.9%)	
4	25 (0.6%)	30 (0.7%)	
Unknown	208 (5.1%)	205 (5%)	
Tumor Size			0.987
≤2 cm	564 (13.8%)	567 (14%)	
2–4 cm	2,499 (61.2%)	2,492 (61%)	
>4 cm	1,018 (25%)	1,022 (25%)	
Stage			0.977
I	312 (7.6%)	314 (7.7%)	
II	3,647 (89.4%)	3,642 (89.2%)	
III	122 (3%)	125 (3.1%)	
T			0.984
T1	155 (3.8%)	160 (4%)	
T2	434 (10.6%)	438 (10.7%)	
T3	3,370 (82.6%)	3,358 (82.3%)	
T4	122 (3%)	125 (3%)	
Nodal status			0.942
Negative	1,219 (30%)	1,216 (30%)	
Positive	2,862 (70%)	2,865 (70%)	

CRT, chemoradiotherapy.

**Figure 2:**
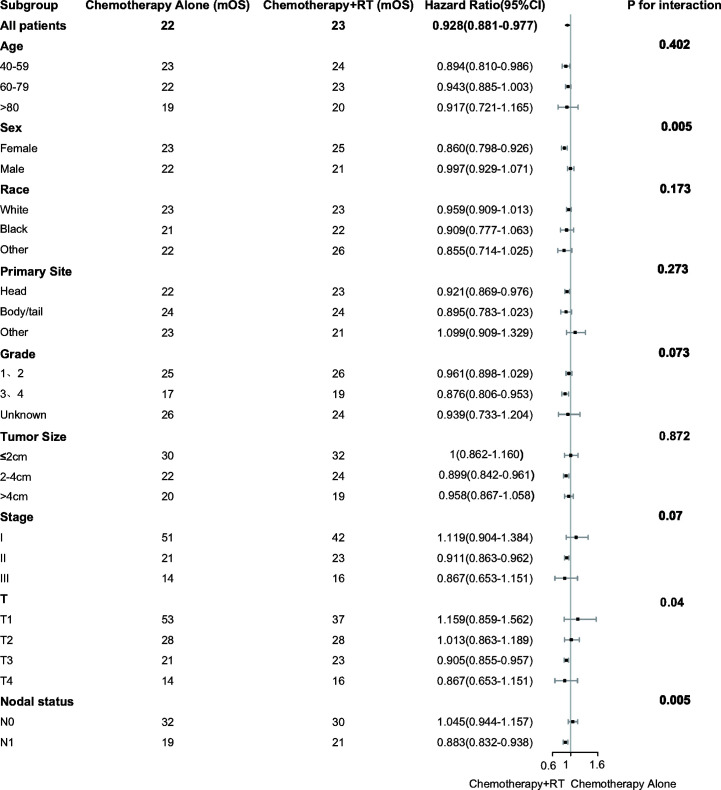
Adjusted overall survival impact of chemotherapy and chemoradiotherapy among subgroups. RT indicates radiotherapy.

Subgroup analysis was performed to determine the clinical features with more significant benefits. It was found that the survival benefit of adjuvant CRT was more significant in female [HR, 0.860 (95% CI, 0.798–0.926) *vs* HR, 0.997 (95% CI, 0.929–1.071); P = 0.005 for interaction]. In the high-risk pathological factors, it was found that the survival benefits of adjuvant CRT were more significant in T3 [HR, 0.905 (95% CI, 0.855–0.957); P = 0.04 for interaction] and lymph nodes positive [HR, 0.883 (95% CI, 0.832–0.938) *vs* HR, 1.045 (95% CI, 0.944–1.157); P = 0.005 for interaction] (**Figure 2**).

## Discussion

Pancreatic cancer is one of the most invasive tumors with poor prognosis. At present, the only cure is surgical resection. However, more than 90% of patients will have local recurrence or distant metastasis after the operation (8, 9, 20). After years of research, it has been proved that postoperative adjuvant therapy can reduce recurrence. However, there is still no consensus on the best adjuvant treatment. Clinical trials of CONKO-001 and the European Study Group for Pancreatic Cancer (ESPAC-1) have demonstrated that adjuvant CT after pancreatic cancer resection can gain survival benefits compared with observation (9, 12, 14). The role of radiotherapy in adjuvant therapy is controversial. The Gastrointestinal Tumor Study Group (GITSG) trials have proved that adjuvant CRT has advantages in survival. However, the subsequent ESPAC-1 and the European Organization for Research and Treatment of Cancer (EORTC) trials did not repeat the results of the GITSG trials, and adjuvant CRT did not show survival benefits compared with the observation (13–15). In this retrospective study, we analyzed 10,092 patients from the SEER database. This research shows that adjuvant CRT does not improve patient survival compared with adjuvant CT without adjusting clinical characteristics. However, adjuvant CRT can improve patient survival after adjustment for confounders in the propensity matching analysis. Subgroup analysis showed that the survival benefit of adjuvant CRT was more significant in patients with females (HR, 0.860; P = 0.005) and T3 (HR, 0.905; P = 0.04) and lymph nodes positive (HR, 0.883; P = 0.005).

There have been many twists and turns in the exploration of adjuvant radiotherapy in the past 30 years. The earliest clinical trial of adjuvant radiotherapy was a small-sample prospective randomized trial conducted by GITSG in 1974. A total of 43 patients with negative incisal margin were randomly divided into two groups: operation + observation group (n = 22) and operation + adjuvant radiotherapy group (n = 21). The results demonstrated that adjuvant CRT can improve the survival rate compared with surgery alone (median OS 11 *vs.* 21.0 months, P = 0.03) (13). After that, 30 patients were non-randomly enrolled in the adjuvant CRT group and achieved the same results (21). Although the trial got positive results, its limitation lies in its small sample size and the use of outdated split course radiotherapy.

To verify the positive results of the GITSG trial, the EORTC III phase trial was carried out subsequently, but the results were not as good as expected. A total of 218 patients were randomly assigned to the observation group (n = 108) and the adjuvant CRT group (n = 110). The results of the pancreatic cancer subgroup study showed that there was no significant difference in survival rate between the observation group and the adjuvant radiotherapy group (2-year survival rate 26 *vs.* 34% P = 0.099) (14). However, the reanalysis showed that the survival rate was statistically significant in a 1-sided analysis (P = 0.049) (22).

This was followed by a larger prospective clinical trial. The ESPAC-1 trial included a total of 541 patients in the 2 × 2 factorial design who were randomly divided into observation and adjuvant CT and observation versus adjuvant CRT. The results showed that adjuvant CT had a better survival rate than surgery alone, whereas adjuvant CRT led to a worse outcome than surgery alone. The study has been criticized for its complexity 2 × 2 factorial design and lack of radiation quality control (11, 15).

Several studies have assessed surveillance, epidemiological, and final results data showing that adjuvant chemotherapy combined with radiotherapy benefits the survival of pancreatic cancer (23–25). A number of studies have used PSM to adjust the effect of adjuvant therapy on the survival of pancreatic cancer. A large retrospective study of a total of 1,092 patients from Johns Hopkins and Mayo Clinic showed that adjuvant CRT had survival benefits compared with surgery alone (median OS 14.3 *vs.* 21.9 months, P < 0.001). Adjuvant CRT can improve the survival rate of patients with negative and positive margins. In addition, lymph nodes positive seemed to have significant benefits in terms of adjuvant CRT while lymph nodes negative did not benefit (26). Several studies based on the National Cancer Database (NCBD) have shown that adjuvant radiotherapy is associated with OS improvement in pancreatic cancer (27–29). Rutter et al. (27) study of 6,165 patients showed that adjuvant CRT was independently associated with OS improvement, especially in patients with R1 resection and N1 disease. An analysis of 12,472 patients from Kamarajah et al. (28) showed that adjuvant radiotherapy can improve survival compared with non-radiotherapy. In further analysis, significant survival benefits were found in patients with positive lymph nodes, but no survival benefits were found in patients with negative lymph nodes. Moaven et al. (29) study included 16,709 patients showed that in the presence of any high-risk pathologic features (nodal or margin involvement or LVI), adjuvant chemotherapy followed by radiation provides a better survival advantage over chemotherapy alone. It was found that radiotherapy alone had no benefit to survival and the importance of treatment sequence. Radiotherapy before chemotherapy was associated with a decline in overall survival, which may be due to delays in systemic treatment. Another single-center small sample retrospective study of 146 patients demonstrated that adjuvant CRT had no significant survival benefit compared with adjuvant CT (median OS 16.8 *vs.* 21.5 months, P = 0.76) (30).

Although no large randomized controlled trials have confirmed the survival benefits of CRT in resectable pancreatic cancer, a large number of retrospective studies have shown that CRT has survival benefits, especially in high-risk patients (lymph nodes positive or cutting edge positive). Our research yielded similar results indicating a more significant benefit of CRT in lymph nodes positive and T3.

There are several limitations to our studies. First of all, this study is a retrospective study of the database, which has treatment and selection bias. This bias is reduced by propensity matching analysis. Secondly, the SEER database does not provide other high-risk information such as lymphatic invasion and marginal status. In addition, the database does not have access to other information about patients, including therapeutic toxicity, physical status, and complications. Finally, the most important limitation is that the database cannot provide accurate information about chemotherapy, including specific chemotherapy regimens and chemotherapy cycles. At the same time, it is impossible to obtain the relevant information about radiotherapy, including the specific dose of radiotherapy, the target area of radiotherapy, the technique of radiotherapy, and the dose of organ at risk.

## Conclusion

All in all, our studies have shown that adjuvant CRT has survival benefits compared with adjuvant CT. The benefit was more significant in patients with female or T3 or lymph nodes positive. More reasonable and optimized prospective randomized clinical trials are needed to further determine the benefits of radiotherapy in resectable pancreatic cancer. In addition, our study provides reference significance for the design of follow-up clinical trials.

## Data Availability Statement

Publicly available datasets were analyzed in this study. This data can be found here: https://seer.cancer.gov/data/.

## Ethics Statement

Ethical approval was not provided for this study on human participants because this study was completely based on the publicly available SEER database and we have got the permission to access them on purpose of research only. It did not include interaction with humans or use personal identifying information. The informed consent was not required for this research.

## Author Contributions

XS and FZ made substantial contributions to the design of the study, carried out the analysis, and interpreted the data. JP and YG contributed to the review of previous literature. HJ and WW contributed substantially to the data discussion and critically commented on the manuscript for scientific content. All authors made substantial contributions to data interpretation and drafting of the manuscript and were responsible for the quality of the overall manuscript. All authors contributed to the article and approved the submitted version.

## Funding

This work was supported by oncology leading discipline construction support project clinical research funding project (No XKJS202005).

## Conflict of Interest

The authors declare that the research was conducted in the absence of any commercial or financial relationships that could be construed as a potential conflict of interest.

## References

[1] Bray F Ferlay J Soerjomataram I Siegel RL Torre LA Jemal A. Global Cancer Statistics 2018: GLOBOCAN Estimates of Incidence and Mortality Worldwide for 36 Cancers in 185 Countries. CA: A Cancer J Clin (2018) 68(6):394–424. 10.3322/caac.21492 30207593

[2] Kamarajah SK Burns WR Frankel TL Cho CS Nathan H. Validation of the American Joint Commission on Cancer (AJCC) 8th Edition Staging System for Patients With Pancreatic Adenocarcinoma: A Surveillance, Epidemiology and End Results (SEER) Analysi. Ann Surg Oncol (2017) 24(7):2023–30. 10.1245/s10434-017-5810-x 28213792

[3] Winter JM Brennan MF Tang LH D'Angelica MI Dematteo RP Fong Y. Survival After Resection of Pancreatic Adenocarcinoma: Results From a Single Institution Over Three Decades. Ann Surg Oncol (2012) 19(1):169–75. 10.1245/s10434-011-1900-3 21761104

[4] Conlon KC Klimstra DS Brennan MF. Long-Term Survival After Curative Resection for Pancreatic Ductal Adenocarcinoma. Clinicopathologic Analysis of 5-Year Survivors. Ann Surg (1996) 223(3):273–9. 10.1097/00000658-199603000-00007 PMC1235115 8604907

[5] Hartwig W Hackert T Hinz U Gluth A Bergmann F Strobel O. Pancreatic Cancer Surgery in the New Millennium: Better Prediction of Outcome. Ann Surg (2011) 254(2):311–9. 10.1097/SLA.0b013e31821fd334 21606835

[6] Hishinuma S Ogata Y Tomikawa M Ozawa I Hirabayashi K Igarashi S. Patterns of Recurrence After Curative Resection of Pancreatic Cancer, Based on Autopsy Findings. J Gastrointest Surg (2006) 10(4):511–8. 10.1016/j.gassur.2005.09.016 16627216

[7] Kim YI Song KB Lee YJ Park KM Hwang DW Lee JH. Management of Isolated Recurrence After Surgery for Pancreatic Adenocarcinoma. Br J Surg (2019) 106(7):898–909. 10.1002/bjs.11144 31162655

[8] Groot VP Rezaee N Wu W Cameron JL Fishman EK Hruban RH. Patterns, Timing, and Predictors of Recurrence Following Pancreatectomy for Pancreatic Ductal Adenocarcinom. Ann Surg (2018) 267(5):936–45. 10.1097/SLA.0000000000002234 28338509

[9] Oettle H Post S Neuhaus P Gellert K Langrehr J Ridwelski K. Adjuvant Chemotherapy With Gemcitabine *vs* Observation in Patients Undergoing Curative-Intent Resection of Pancreatic Cancer: A Randomized Controlled Trial. JAMA (2007) 297(3):267–77. 10.1001/jama.297.3.267 17227978

[10] Neoptolemos JP Stocken DD Bassi C Ghaneh P Cunningham D Goldstein D. Adjuvant Chemotherapy With Fluorouracil Plus Folinic Acid *vs* Gemcitabine Following Pancreatic Cancer Resection: A Randomized Controlled Trial. JAMA (2010) 304(10):1073–81. 10.1001/jama.2010.1275 20823433

[11] Neoptolemos JP Stocken DD Friess H Bassi C Dunn JA Hickey H. A Randomized Trial of Chemoradiotherapy and Chemotherapy After Resection of Pancreatic Cancer. New Engl J Med (2004) 350(12):1200–10. 10.1056/NEJMoa032295 15028824

[12] Oettle H Neuhaus P Hochhaus A Hartmann JT Gellert K Ridwelski K. Adjuvant Chemotherapy With Gemcitabine and Long-Term Outcomes Among Patients With Resected Pancreatic Cancer: The CONKO-001 Randomized Trial. JAMA (2013) 310(14):1473–81. 10.1001/jama.2013.279201 24104372

[13] Kalser MH Ellenberg SS. Pancreatic Cancer. Adjuvant Combined Radiation and Chemotherapy Following Curative Resection. Arch Surg (1985) 120(8):899–903. 10.1001/archsurg.1985.01390320023003 4015380

[14] Klinkenbijl JH Jeekel J Sahmoud T van Pel R Couvreur ML Veenhof CH. Adjuvant Radiotherapy and 5-Fluorouracil After Curative Resection of Cancer of the Pancreas and Periampullary Region: Phase III Trial of the EORTC Gastrointestinal Tract Cancer Cooperative Group. Ann Surg (1999) 230(6):776–82; discussion 82-4. 10.1097/00000658-199912000-00006 10615932 PMC1420941

[15] Neoptolemos JP Dunn JA Stocken DD Almond J Link K Beger H. Adjuvant Chemoradiotherapy and Chemotherapy in Resectable Pancreatic Cancer: A Randomised Controlled Trial. Lancet (London England) (2001) 358(9293):1576–85. 10.1016/s0140-6736(01)06651-x 11716884

[16] Koshy MC Landry JC Cavanaugh SX Fuller CD Willett CG Abrams RA. A Challenge to the Therapeutic Nihilism of ESPAC-1. Int J Radiat Oncol Biol Phys (2005) 61(4):965–6. 10.1016/j.ijrobp.2004.11.018 15752874

[17] Garofalo M Flannery T Regine W. The Case for Adjuvant Chemoradiation for Pancreatic Cancer. Best Pract Res Clin Gastroenterol (2006) 20(2):403–16. 10.1016/j.bpg.2005.11.001 16549335

[18] Abrams RA Lillemoe KD Piantadosi S. Continuing Controversy Over Adjuvant Therapy of Pancreatic Cancer. Lancet (London England) (2001) 358(9293):1565–6. 10.1016/S0140-6736(01)06666-1 11716876

[19] Choti MA. Adjuvant Therapy for Pancreatic Cancer–the Debate Continues. New Engl J Med (2004) 350(12):1249–51. 10.1056/NEJMe048002 15028829

[20] Zhang Y Frampton AE Kyriakides C Bong JJ Habib N Ahmad R. Loco-Recurrence After Resection for Ductal Adenocarcinoma of the Pancreas: Predictors and Implications for Adjuvant Chemoradiotherapy. J Cancer Res Clin Oncol (2012) 138(6):1063–71. 10.1007/s00432-012-1165-7 PMC11824181 22392075

[21] Further Evidence of Effective Adjuvant Combined Radiation and Chemotherapy Following Curative Resection of Pancreatic Cancer. Gastrointestinal Tumor Study Grou. Cancer (1987) 59(12):2006–10. 10.1002/1097-0142(19870615)59:12<2006::aid-cncr2820591206>3.0.co;2-b 3567862

[22] Garofalo MC Regine WF Tan MT. On Statistical Reanalysis, the EORTC Trial is a Positive Trial for Adjuvant Chemoradiation in Pancreatic Cancer. Ann Surg (2006) 244(2):332–3; author reply 3. 10.1097/01.sla.0000229980.81505.44 16858208 PMC1602155

[23] Hazard L Tward JD Szabo A Shrieve DC. Radiation Therapy is Associated With Improved Survival in Patients With Pancreatic Adenocarcinoma: Results of a Study From the Surveillance, Epidemiology, and End Results (SEER) Registry Data. Cancer (2007) 110(10):2191–201. 10.1002/cncr.23047 17918259

[24] Artinyan A Hellan M Mojica-Manosa P Chen YJ Pezner R Ellenhorn JD. Improved Survival With Adjuvant External-Beam Radiation Therapy in Lymph Node-Negative Pancreatic Cancer: A United States Population-Based Assessment. Cancer (2008) 112(1):34–42. 10.1002/cncr.23134 18000805

[25] McDade TP Hill JS Simons JP Piperdi B Ng SC Zhou Z. A National Propensity-Adjusted Analysis of Adjuvant Radiotherapy in the Treatment of Resected Pancreatic Adenocarcinoma. Cancer (2010) 116(13):3257–66. 10.1002/cncr.25069 20564625

[26] Hsu CC Herman JM Corsini MM Winter JM Callister MD Haddock MG. Adjuvant Chemoradiation for Pancreatic Adenocarcinoma: The Johns Hopkins Hospital-Mayo Clinic Collaborative Study. Ann Surg Oncol (2010) 17(4):981–90. 10.1245/s10434-009-0743-7 PMC2840672 20087786

[27] Rutter CE Park HS Corso CD Lester-Coll NH Mancini BR Yeboa DN. Addition of Radiotherapy to Adjuvant Chemotherapy Is Associated With Improved Overall Survival in Resected Pancreatic Adenocarcinoma: An Analysis of the National Cancer Data Bas. Cancer (2015) 121(23):4141–9. 10.1002/cncr.29652 26280559

[28] Kamarajah SK Sonnenday CJ Cho CS Frankel TL Bednar F Lawrence TS. Association of Adjuvant Radiotherapy With Survival After Margin-Negative Resection of Pancreatic Ductal Adenocarcinoma: A Propensity-Matched National Cancer Database (NCDB) Analysi. Ann Surg (2019) 273(3):587–94. 10.1097/SLA.0000000000003242 30817352

[29] Moaven O Clark CJ Russell GB Votanopoulos KI Howerton R Levine EA. Optimal Adjuvant Treatment Approach After Upfront Resection of Pancreatic Cancer: Revisiting the Role of Radiation Based on Pathologic Feature. Ann Surg (2020). 10.1097/SLA.0000000000003770 PMC7335684 31913868

[30] Martin LK Luu DC Li X Muscarella P Ellison EC Bloomston M. The Addition of Radiation to Chemotherapy Does Not Improve Outcome When Compared to Chemotherapy in the Treatment of Resected Pancreas Cancer: The Results of a Single-Institution Experience. Ann Surg Oncol (2014) 21(3):862–7. 10.1245/s10434-013-3266-1 PMC4041711 24046122

